# Abnormal reward prediction-error signalling in antipsychotic naive individuals with first-episode psychosis or clinical risk for psychosis

**DOI:** 10.1038/s41386-018-0056-2

**Published:** 2018-04-05

**Authors:** Anna O. Ermakova, Franziska Knolle, Azucena Justicia, Edward T. Bullmore, Peter B. Jones, Trevor W. Robbins, Paul C. Fletcher, Graham K. Murray

**Affiliations:** 1 Department of Psychiatry, University of Cambridge, Cambridge, UK; 2 Behavioural and Clinical Neuroscience Institute, University of Cambridge, Cambridge, UK; 3 Cambridgeshire and Peterborough NHS Foundation Trust, Cambridge, UK; 4 Department of Psychology, University of Cambridge, Cambridge, UK; 5 Institute of Metabolic Science, University of Cambridge, Cambridge, UK

## Abstract

Ongoing research suggests preliminary, though not entirely consistent, evidence of neural abnormalities in signalling prediction errors in schizophrenia. Supporting theories suggest mechanistic links between the disruption of these processes and the generation of psychotic symptoms. However, it is unknown at what stage in the pathogenesis of psychosis these impairments in prediction-error signalling develop. One major confound in prior studies is the use of medicated patients with strongly varying disease durations. Our study aims to investigate the involvement of the meso-cortico-striatal circuitry during reward prediction-error signalling in earliest stages of psychosis. We studied patients with first-episode psychosis (FEP) and help-seeking individuals at-risk for psychosis due to sub-threshold prodromal psychotic symptoms. Patients with either FEP (*n* = 14), or at-risk for developing psychosis (*n* = 30), and healthy volunteers (*n* = 39) performed a reinforcement learning task during fMRI scanning. ANOVA revealed significant (*p* < 0.05 family-wise error corrected) prediction-error signalling differences between groups in the dopaminergic midbrain and right middle frontal gyrus (dorsolateral prefrontal cortex, DLPFC). FEP patients showed disrupted reward prediction-error signalling compared to controls in both regions. At-risk patients showed intermediate activation in the midbrain that significantly differed from controls and from FEP patients, but DLPFC activation that did not differ from controls. Our study confirms that FEP patients have abnormal meso-cortical signalling of reward-prediction errors, whereas reward-prediction-error dysfunction in the at-risk patients appears to show a more nuanced pattern of activation with a degree of midbrain impairment but preserved cortical function.

## Introduction

The cognitive basis of psychotic symptoms remains unknown, but abnormalities in the processing of prediction error have been proposed to contribute to the development of psychotic symptoms [1, 2]. A prediction error is the discrepancy between something we expect to happen (e.g. receiving a reward after a chosen stimulus), based on experience (e.g. we have received a reward after selecting this stimulus on prior occasions), and what actually happens (e.g. no reward is provided). Prediction errors help us to update our expectations and can lead to allocation of attention and attribution of salience to stimuli, which may drive subsequent learning [3, 4]. Faulty prediction-error signalling could lead to several maladaptive psychological processes that have been proposed to contribute to the generation of psychotic symptoms: aberrant assignment of attention and motivational importance to innocuous stimuli, and disrupted associative learning leading to the formation of irrelevant associations and eventually delusions [5–9].

Several studies have attempted to examine the neural basis of prediction-error abnormalities in psychosis, and have documented blunted midbrain, striatal and/or cortical encoding of reward prediction errors [10–13] and non-reward-related prediction errors [14]. Our previous work in psychosis patients has identified meso-cortico-striatal prediction-error deficits, involving midbrain, striatum and frontal cortex, especially right dorsolateral prefrontal cortex (DLPFC) [12, 14]. Dopaminergic neurons in the midbrain, which project heavily to the striatum as well as to the cortex, have been found to code reward prediction errors [15]. Meso-cortical-striatal regions, including the right DLPFC, are activated during associative learning in functional Magnetic Resonance Imaging (fMRI) studies, especially when expectations are violated, and the fMRI signal scales with prediction-error magnitude [16–19]. Work from our group and others has supported this view by showing differences in the right DLPFC between patients and controls [12, 18, 20]. Dysfunction of these regions may manifest in abnormalities in learning and motivational salience [21–23], potentially contributing to the development of psychosis [24]. However, a major complication in the interpretation of patient studies is that several studies are potentially confounded by having either all, or the majority of patients, taking antipsychotic medication, which has been shown to modulate brain-reward processing in healthy individuals and patients [25–27]. Given this, and the likely importance of dopaminergic dysfunction in the pathogenesis of psychosis [28], it is critical to investigate possible abnormalities during reward prediction-error processing in antipsychotic naive patient samples. To our knowledge, only two studies [13, 29] have examined reward prediction-error signalling in unmedicated, but not all antipsychotic naive, samples of mixed first episode and chronic schizophrenia patients (average age 27 years [13], average age 34 years [29]). Although both of these studies document striatal reward prediction-error abnormalities, neither study report abnormalities in the dopaminergic midbrain. This is of particular interest given that the extensive evidence for the role of dopamine in both prediction-error signalling [15, 30] and the pathophysiology of psychosis.

Some studies of chronic medicated schizophrenia patients have shown intact prediction error-associated brain signals [10, 31, 32]. It is possible that these neural abnormalities, as well as related behavioural manifestations of altered learning, may be more prominent early in the course of the illness, especially in antipsychotic naive samples [5, 12, 13, 29]. Establishing the pathophysiological abnormalities at the very earliest stages of illness is likely to be critical for optimal treatment and preventative interventions. The onset of psychosis is usually preceded by a prodromal phase involving social, educational or occupational decline accompanied by prodromal symptoms such as suspiciousness or hallucinations without a delusional interpretation [33]. Help-seeking patients with these features have been shown to have increased risk for developing psychotic illness, and have been termed to have at-risk mental states (ARMS) or be at “ultra-high clinical risk” of psychosis [33]. The prodromal phase may offer a critical period for intervention to improve long-term prognosis. The study of at-risk patients has proved a useful paradigm to investigate some of the earliest pathophysiological changes in schizophrenia and related illness [34]. Brain prediction-error signalling has not been examined in this group before, although there is some evidence for abnormal cortical and/or striatal processing of salience [35, 36] or reward anticipation in at-risk patients [37, 38]. Given the theoretical importance of prediction error in learning and the pathogenesis of symptoms, we reasoned that it is key to examine brain prediction-error signals in the earliest possible stages of psychosis. In this study, we set out to examine fMRI-correlates of reward prediction errors in a sample of patients with first-episode pyschosis (FEP) and in at-risk patients, all naive to antipsychotic medication, with a particular focus on the midbrain, striatum and right DLPFC.

A simple view of the continuum of psychosis is that at-risk patients with sub-threshold symptoms will show similar pathology to clinical psychosis but of lesser severity [39] and there is evidence in support of this [34]. We therefore hypothesised that patients with FEP would have abnormal prediction-error activity in the dopaminergic midbrain, striatum and right DLPFC compared to controls, and that at-risk patients would have brain prediction-error activation patterns intermediate between FEP patients and controls.

## Methods

### Participants

The study was approved by the Cambridgeshire three National Health Service research ethics committee. Individuals with FEP (*n* = 14, average 23.57 years, 7 female) or at-risk for psychosis individuals (*n* = 30, average 22.03 years, 14 female) were recruited from the Cambridgeshire first-episode psychosis service, CAMEO. Inclusion criteria were as follows: age 16–35 years, early psychosis as reflected by meeting either at-risk attenuated psychotic symptoms criteria according to the Comprehensive Assessment of At-risk Mental States (CAARMS, [33]) or FEP criteria. Patients with FEP were required to meet ICD-10 criteria for a schizophrenia spectrum disorder (F20, F22, F23, F25, F28, F29) or affective psychosis (F30.2, F31.2, F32.3), to be within 1 year of first presentation to the clinical service for psychosis, and to have ongoing positive psychotic symptoms (see Supplementary Material for details of diagnostic breakdown in the FEP group); all participants were required to be naive to antipsychotic medication. Healthy volunteers (*n* = 39, average 23.23 years, 20 female) without a history of psychiatric illness or brain injury were recruited as control subjects. All subjects had normal or corrected-to-normal vision and no contraindications to MRI-scanning. None of the participants had a recreational drug or alcohol dependence. Healthy volunteers did not report any personal or family history of significant neurological, psychiatric or medical disorders, and were matched to patients regarding age, gender, handedness and maternal level of education. There were no significant differences between groups in age, sex, handedness or recreational drug use (Table 1). Antidepressant medication was taken by one control (sertraline), four FEP (two fluoxetine and two sertraline) and eight ARMS (two fluoxetine, two citalopram, one sertraline, one amitriptyline (low dose), two unknown). The use of antidepressants differed significantly between controls and patients (Table 1, see supplements for description of effect of antidepressant on behavioural performance and imaging results). We used the Culture Fair matrices test, which is a general IQ measure. There was a significant difference between groups in IQ. The three groups were not intended to be fully matched on IQ, as cognitive impairment is common in psychosis, and the task is not intellectually taxing. However, the groups were matched in maternal education, which indicates intellectual potential was matched. Even though the groups slightly differed in IQ, we did not find group differences in the performance. Furthermore, we did not find significant correlations between performance and IQ in any of the trial types. Therefore, we are confident that the group difference observed in our sample is due to psychiatric differences rather than differences in IQ. On average, the patients had predominantly positive psychotic symptoms (it was an entry requirement to have some degree of current positive symptoms) and low levels of negative symptoms. FEP had significantly more severe symptoms than the at-risk group. Use of alcohol and other drugs was measured on a five-point scale from (Table 1). The control subjects, a typical group of healthy young adults, tend to drink slightly more alcohol than the patients. Our clinical experience suggests that patients from our service, many of whom have paranoia and/or social anxiety, and some of whom have negative symptoms, socialise less frequently than controls. This may partly explain why the patient groups consume less alcohol. No significant correlations between use of alcohol and performance or brain signalling were detected. Written informed consent was supplied by all participants.

**Table 1:** Sample characteristics for healthy controls, for participants at-risk and with FEP

	At-risk		FEP		Controls		Statistical test results
	Mean	SD	Mean	SD	Mean	SD	
Age	22.03	3.30	23.57	5.80	23.23	3.53	*F*(2,82) = 1.07, *p* = 0.35
IQ	105.53	13.86	103.46	15.88	113.47	11.15	*F*(2,80) = 4.48, *p* = 0.014
Mother’s education	1.80	1.56	2.43	1.79	2.18	1.41	*F*(2,82) = 0.94, *p* = 0.39
Gender (m/f)	16/14		7/7		19/20		*χ*^2^ (2) = 0.15, *p* = 0.93
Handedness (r/h)	26/4		10/4		35/4		*χ*^2^ (2) = 2.84, *p* = 0.24
Smoking (yes/no)	15/15		9/5		12/27		*χ*^2^ (2) = 5.55, *p* = 0.06
Antidepressants (yes/no)	8/30		4/14		1/39		*χ*^2^ (2) = 9.58, *p* = 0.008
	*Mean*	*SD*	*Mean*	*SD*	*Mean*	*SD*	*Statistical test results*
Alcohol	2.53	1.00	1.50	1.45	2.59	0.68	*F*(2,82) = 7.13, *p* = 0.001
Cannabis	1.03	1.12	1.36	1.60	0.95	0.97	*F*(2,82) = 0.65, *p* = 0.52
Hallucinogens	0.30	0.53	0.29	0.47	0.28	0.56	*F*(2,82) = 0.01, *p* = 0.99
Stimulants	0.50	0.63	0.71	0.82	0.46	0.79	*F*(2,82) = 0.61, *p* = 0.55
Depressants/opiates	0.13	0.43	0.07	0.27	0.10	0.38	*F*(2,81) = 0.13, *p* = 0.88
BDI	24.86	14.06	26.45	8.20			*T* = 0.35, df = 37, *p* = 0.73
PANSS positive	11.07	3.17	16.07	4.92			*T* = 4.4, df = 42, *p* < 0.001
PANSS negative	9.03	3.71	9.29	4.77			*T* = 0.19, df = 42 *p* = 0.85
CAARMS total psychosis score	15.10	6.75	23.86	6.48			*T* = 4.1, df = 42, *p* < 0.001
UTC	2.0	2.08	3.71	2.46			*T* = 2.39, df = 42, *p* = 0.02
UTC Freq	1.76	1.92	3.21	2.15			*T* = 2.23, df = 42, *p* = 0.03
NBI	3.10	1.37	4.64	1.64			*T* = 3.26, df = 42, *p* = 0.002
NBI Freq	3.13	1.33	4.00	1.41			*T* = 1.97. df = 42, *p* = 0.06
PA	2.83	1.82	4.57	1.50			*T* = 3.11, df = 42, *p* = 0.001
PA Freq	2.27	1.68	3.71	1.49			*T* = 2.76, df = 42, *p* = 0.009

Psychopathology scales were compared across the patient groups only. Use of alcohol and other drugs was measured on a five-point scale (alcohol use: 0—none, 1—not more than three times, 2—occasional user, 3—regular user (1–3 times weekly), 4—frequent user (almost every day)); drug use: 0—never tried, 1—not more than three times, 2—occasional user, 3—regular user (1–3 times weekly), 4—frequent user (almost every day)

Comprehensive Assessment of At-Risk Mental States (CAARMS) subscales: Unusual Thought Content (UTC), Non-Bizarre Ideas (NBI), Perceptual Abnormalities (PA); total psychosis on CAARMS was calculated as the sum of the intensity and frequency of UTC, NBI and PA subscales. Statistical tests were conducted across three groups for the demographic variables and substance use. The two clinical groups were compared on psychiatric groups

*BDI* Beck Depression Inventory, *GAF* Global Assessment of Functioning, *f* frequency, *Χ*^2^ Pearson’s chi-square, *F* ANOVA *F*-statistic, *H* Kruskal–Wallis test statistic, *SD* standard deviation

### fMRI reward task

**Fig. 1:**
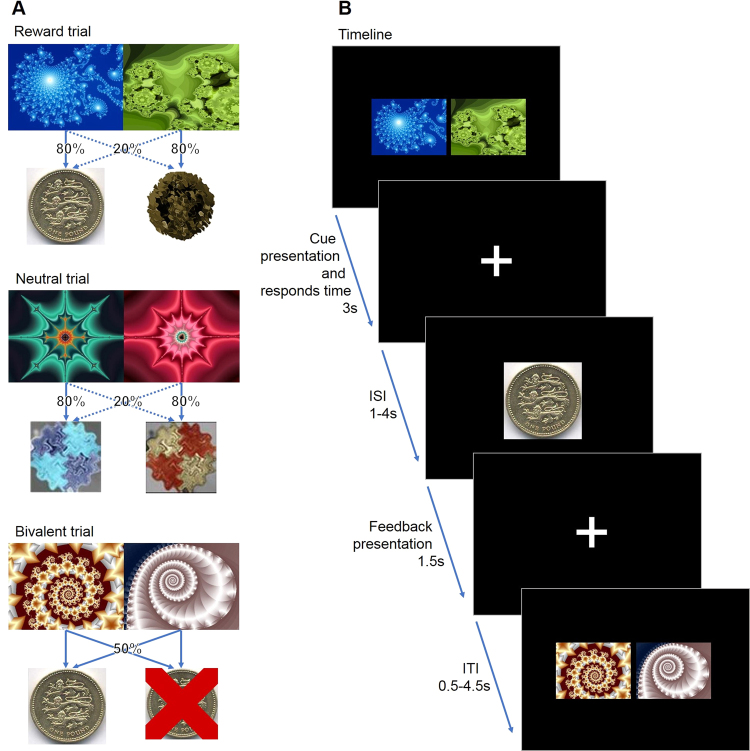
Behavioural task. **a** The three different trial types and feedback probabilities. **b** The experimental task, including trial timing

During the fMRI-scan, participants performed a probabilistic monetary learning task (Fig. 1) that required them to choose between two abstract visual stimuli (fractal pictures) displayed on a computer screen, to maximise pay-offs [12, 26, 40, 41]. On each trial, the participant chose one of two stimuli, then feedback was provided. From the feedback, the participant learnt which of the pictures were more likely to give a reward of £1, or a loss of £1, and which ones were neutral. Each one of the three pairs of stimuli were presented in 30 trials (90 randomised trials in total per subject). The stimuli within each pair led to a specific outcome with different probabilities: Reward trials: one picture led to a £1 win in 80% of trials and to neutral feedback in 20% of trials, the other picture led to a neutral outcome in 80% of trials and to a £1 win in 20% of trials; bivalent trials: there was a 50% chance of either losing or winning £1 [41]; neutral trials: 80%/20% chance of receiving two kinds of neutral feedback (Fig. 1a). The order of the pictures presented and the position of the high-probability stimulus were counterbalanced across trials of the same valence and pseudo-randomised. To win money, the participants had to learn by trial and error, to learn which stimulus was more likely to produce a reward. The participants were informed that any money won during the experiment would be paid to them at the end of the study.

### Behavioural analysis

A 3×3 mixed-model analysis of variance (ANOVA, group × trial type) was used to investigate the group differences in reaction times and stimuli choices in the three types of trials (reward, bivalent and neutral). We examined the proportion of “correct” responses. Here the term “correct” means selecting the picture that leads to a high probability of getting £1 in the reward trial, and the picture with the higher probability of receiving the blue feedback picture in neutral trials, and is randomly assigned to choosing one of the pictures in bivalent trials (Fig. 1a). In the bivalent and neutral trials, the assignment of “correctness” is arbitrary, but assigning one stimulus in each category to be the “correct” stimulus allows examination of whether participants preferred one stimulus over another and the extent to which response patterns differed across trial types.

### fMRI data acquisition and analysis

Full details of our 3T scanning protocol and analysis methods are available in the Supplementary Material.

The seven explanatory variables (regressors) that we used were as follows: (1) onset of the bivalent cues; (2) onset of the neutral cues; (3) onset of the reward cues; (4) neutral outcome onsets (neutral feedback) during both neutral and reward trials; (5) winning outcome onset in the reward trials; (6) winning outcome onset in the bivalent trials; (7) loss outcome onset in bivalent trials. All regressors were modelled as 2 s events and convolved with a canonical double-gamma response function. We added temporal derivatives to the model to account for possible variation in the haemodynamic response function and we included motion parameters.

Our contrast of interest aimed at detecting activation associated with positive prediction error, and follows a contrast originally used by Seymour and colleagues who employed a similar paradigm in a healthy volunteer study to examine positive prediction error [41]. We contrasted winning £1 in bivalent trials versus winning £1 in reward trials; contrasting these identical outcomes in the context of different expectations represents a measure of positive prediction error. On the reward trials the reward is well predicted and elicits a low, yet still positive, prediction error; however, the outcome is unpredictable on bivalent trials and hence elicits a high positive prediction error [41]. Therefore, contrasting the two events gives a measure of high versus low prediction error, and hence provides an assay of prediction-error brain activation. This prediction-error contrast has the advantage that it is perfectly balanced in terms of outcome value; hence it is unconfounded by reward outcome value or valence, which has been proposed to be a potential confound of alternative approaches, particularly in designs where reward prediction error and reward value are collinear [42, 43].

For the group ANOVA analysis, we used this prediction-error contrast of interest as the outcome variable (FSL software calls outcome variables COPES: contrast of parameter estimates) and group as the predictor variable. We used permutation based statistics using the FSL tool randomise, utilising threshold-free-cluster enhancement, which enhances cluster-like structures but remains fundamentally a voxel-wise statistical testing method [44]. We report results at *p* = 0.05 or less, family-wise error corrected for multiple comparisons, using the variance smoothing option (3 mm) as recommended for experiments with modest sample sizes, as is common in fMRI research [45]. Our main analysis was based on a region of interest approach as follows. Our primary region of interest was the dopaminergic midbrain using the probabilistic atlas [46], in which traditional anatomical segmentation was replicated using a seed-based functional connectivity approach and which provides a mask that includes substantia nigra and ventral tegmental area. The probabilistic map used to assess midbrain activation has been reliably used in a number of studies [47, 48]. In our two secondary regions of interest, we investigated the associative and limbic striatum (using a single hand-drawn mask, encompassing both associative and limbic striatum, based on operational criteria [49, 50]), and the right DLPFC (utilising a sphere, 10 mm, centred at *x* = 50, *y* = 30, *z* = 28, based on our previous work [14]. Supplementary Figure 1 shows our primary and secondary regions of interest. As ANOVA will show whether the groups deviate from each other, it does not show the direction of effects. We therefore planned that for any voxels deemed significant in the ANOVAs, we would proceed to planned paired group comparisons, again using FSL randomise to test our hypothesis that on the contrast of interest, the following pattern would be seen: controls > at-risk > FEP). For each individual, we also extracted contrast values (contrast of parameter estimates, or COPEs in FSL) from voxels in which significant group differences were found, and calculated cluster averages; the extracted values were used for correlations with symptoms (within group), for bar charts of group differences and, as a supplement to our randomise paired group tests, to further test our hypothesis of controls > at-risk > FEP.

In support of this initial analysis and results, we also ran an additional analysis with a general linear model where a prediction-error regressor was determined by a computational *Q*-learning model as we and others have used previously [12, 26, 40] (methods and results reported in Supplementary Material).

## Results

### Behaviour results: choices

**Fig. 2:**
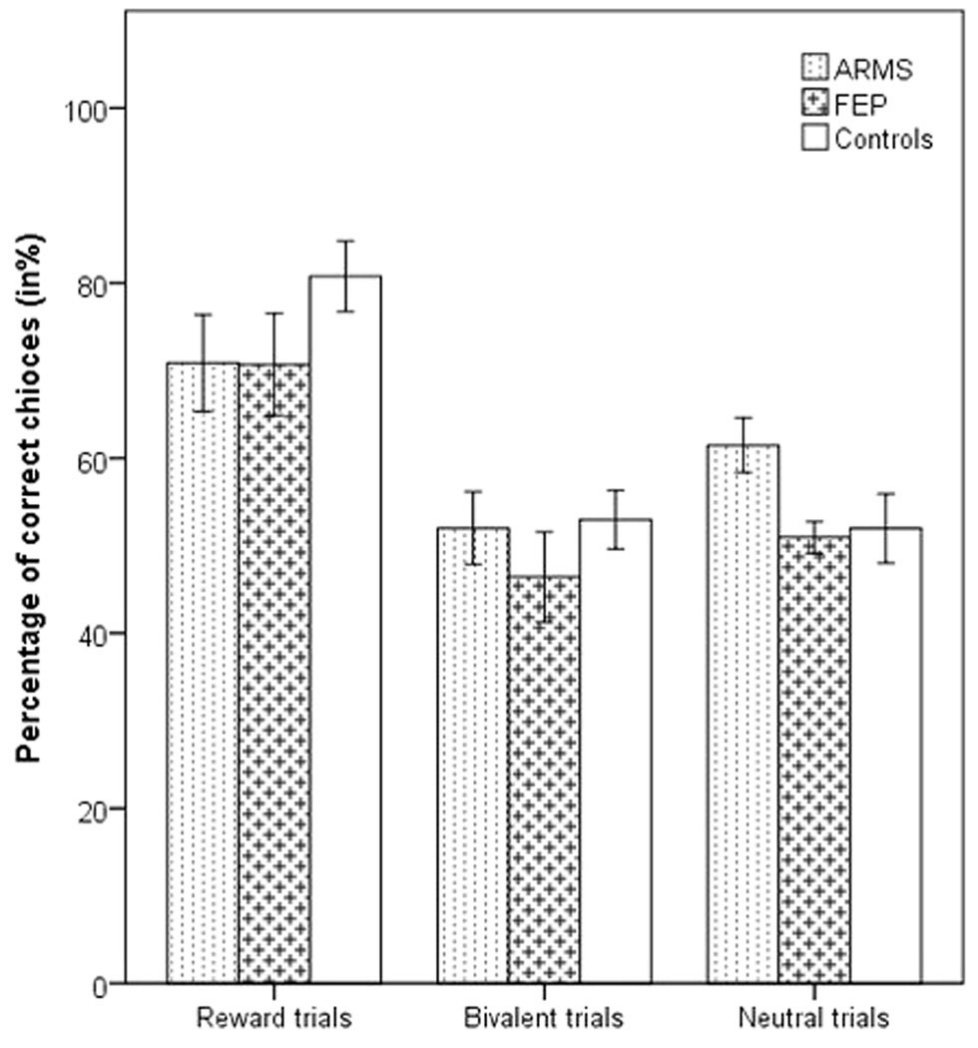
Trial performance: percentage of “correct” (high-likelihood) choices stratified by trial type and participant group. Error bars are ± 1 SE

On analysis of “correct” choices across trial type and group, there was a main effect of trial type (*F* = 20.93, df = 2.160, *p* < 0.001), but no effect of group (*F* = 1.10, df = 2.80, *p* = 0.34) or group by trial type interaction (*F* = 1.53, df = 4.160, *p* = 0.20; see Fig. 2). The probability of choosing the “correct” stimulus increased in the course of the experiment (for a display of learning curves for each condition see Supplementary Figure 2A–C). Bonferroni-corrected post-hoc tests showed that participants chose the “correct” stimulus more frequently in reward trials than in neutral (*p* < 0.001), or bivalent (*p* < 0.001), with no difference between neutral and bivalent trials (*p* = 0.7). Participants learned to choose the picture that most often led to winning £1 (high-probability stimulus: “correct” response) in the majority of reward trials (mean percentage of “correct” choices 75%), whereas performance on other trials was similar to chance (neutral trials 55%, bivalent trials 52%).

To investigate whether participants used the feedback to make an informed decision, further demonstrating engagement in the task, we conducted a win-stay lose-shift analysis (Supplementary Figure 3). We conducted a repeated measure ANOVA with 2 (win stay, lose shift) × 3 (reward, bivalent, neural) analysis across groups. Across all trial types and groups, we found a significantly higher stay probability after a rewarded (in the case of neutral: colour matching) trial compared to lose-shifting on unrewarded (non-matching) trials (win stay: 69.01% ± 1.62, lose shift: 43.69% ± 1.70; *F* = 75.56, df = 1.80, *p* < 0.001). There was a significant trial type effect (*F* = 6.0, df = 2, *p* = 0.003), as well as win stay/lose shift by group interaction (*F* = 8.40, df = 1.2, *p* < 0.001), a win stay/lose shift by trial type interaction (*F* = 32.79, df = 1.2, *p* < 0.001), and a marginally significant trial type by group interaction (*F* = 2.26, df = 2.2, *p* = 0.065).

In our planned group comparisons (Supplementary Table 1 and Supplementary Figure 3), we found that controls had a significantly higher probability for a win stay behaviour than FEP patients on reward and neutral trial types (reward trials: *p* = 0.027; neutral trials: *p* = 0.012) and marginally on bivalent trials (*p* = 0.062). Controls and at-risk patients were similarly likely to repeat the same response after a win. At-risk patients had a significantly higher probability of win stay behaviour in neutral trials compared to FEP (*p* = 0.013), but the two patient groups did not differ on the other trial types. FEP patients had a higher probability for a lose shift behaviour compared to controls and at-risk patients in both reward and neutral trials (reward trials: FEP > controls: *p* = 0.001, FEP > at-risk: *p* = 0.008; neutral trials: FEP > controls: *p* = 0.001, FEP > at-risk: *p* = 0.045). Controls and at-risk patients were similarly likely to shift after a loss.

### Behaviour results: reaction times

We analysed reaction times trial type and group. We found a significant main effect of trial type (*F* = 4.473, df = 2.160, *p* = 0.01), but no effect of group (*F* = 0.32, df = 2.80, *p* = 0.73) or group by trial type interaction (*F* = 1.28, df = 4.160, *p* = 0.28). The Bonferroni-corrected post-hoc tests revealed that participants independent of group reacted significantly (*p* < 0.001) faster to reward trials (1122.83 ms ± 33.31) than to bivalent trials (1358.99 ms ± 41.49), and similarly (*p* = 0.14) to neutral trials (1329.30 ms ± 97.87).

### Prediction-error imaging results: ANOVA across three groups

We conducted second level (i.e. group level) ANOVAs using FSL randomise across the three groups across the whole brain and in our region of interests. Our outcome measure presented here in the main manuscript text is the contrast value (in FSL termed contrast of parameter estimates, or COPE) of a bivalent trial win versus a reward trial win, which corresponds to positive reward prediction error; group is the predictor variable. Results from a related outcome variable—prediction error-associated brain activity derived from the computationally modelled prediction error—are presented in the Supplementary Material, and are similar. On whole-brain analysis, there were no group differences that passed our statistical threshold corrected for multiple comparisons.

### Primary region of interest results: prediction-error imaging results in the dopaminergic midbrain

**Fig. 3:**
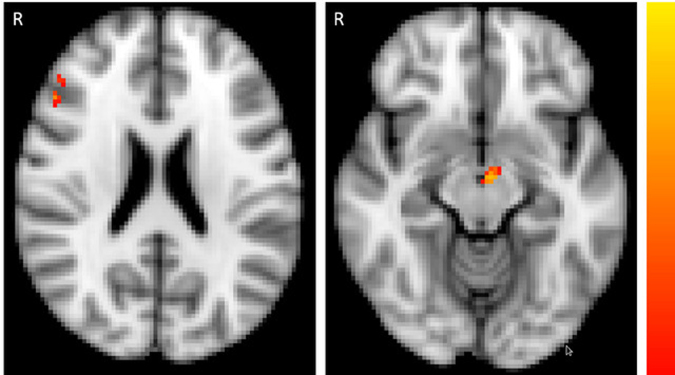
Group differences in region of interest analysis of activation associated with reward prediction-error signal (*p* < 0.05 FWE-corrected) across the three groups (controls, first-episode psychosis and at-risk patients) in the midbrain ventral tegmental area (right panel, *z* = −12), and right middle gyrus/dorsolateral prefrontal cortex (left panel, *z* = 22). Colour bar depicts corrected voxel *p*-value from 0.001 (yellow) to 0.05 (red)

**Fig. 4:**
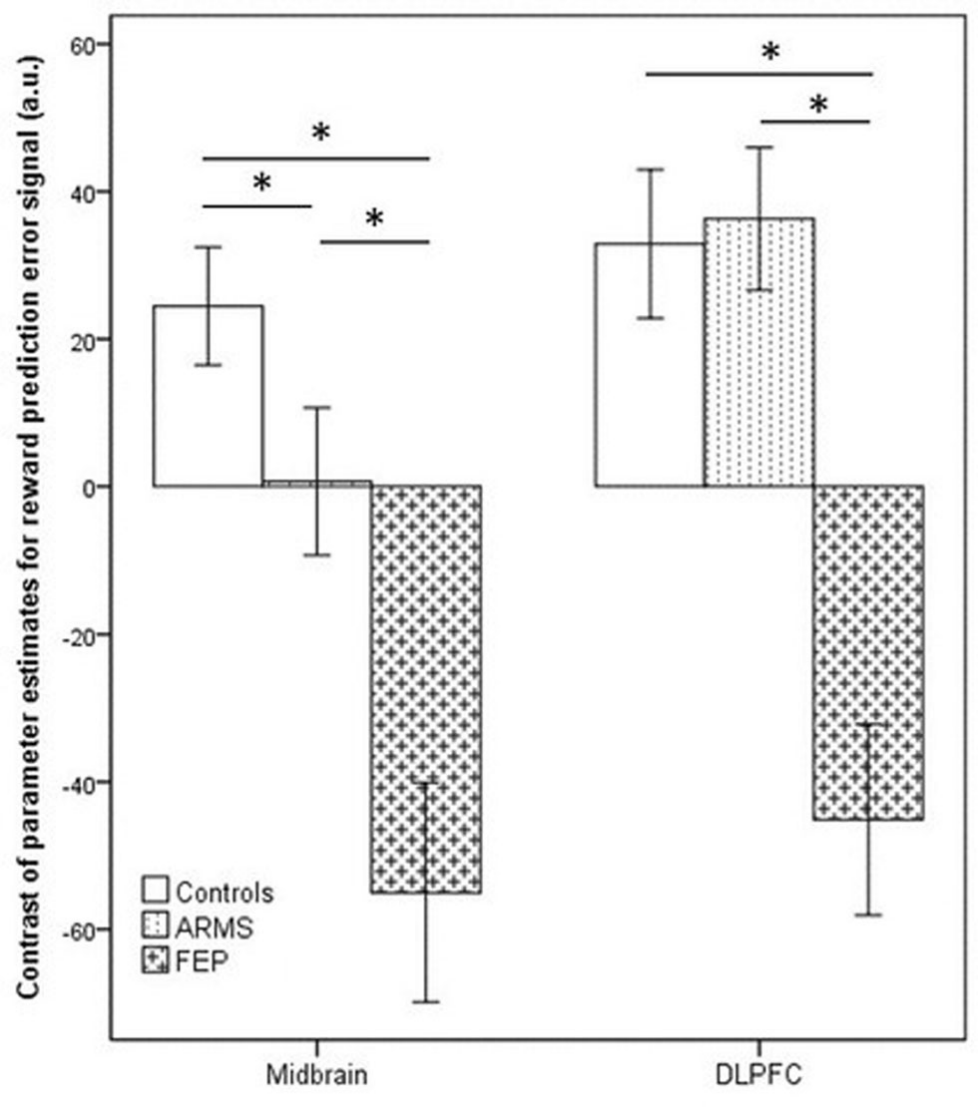
Bar chart shows the mean prediction-error contrast values (termed contrast of parameter estimates, or COPEs in FSL) according to the group, extracted from the significant clusters determined by FSL randomised ANOVA results. The contrast values (COPEs) are derived from the contrast between Reward win and Bivalent win, which constitute the reward prediction error. Error bars show ± 1 SE; a.u. arbitrary units. *p < 0.05

We found a significant family-wise-error corrected main effect for group in the primary region of interest, the dopaminergic midbrain (maximal difference at *x* = −4, *y* = −12, *z* = −12; *t* = 3.45, *p* = 0.013 FWE-corrected, 39 voxels; Fig. 3). For each of these 39 significant voxels, family-wise error correcting for multiple comparisons, we performed planned group comparisons between pairs of groups using randomise to test our hypothesis of controls > at-risk patients > FEP patients. The results were consistent with the hypothesis (Fig. 4): at-risk patients were intermediate and significantly differed from both FEP patients and controls (controls > at-risk patients maximal difference at *x* = 0, *y* = −16, *z* = −8; *t* = 2.86, *p* = 0.033 FWE-corrected, 3 voxels; at-risk > FEP patients, maximal difference at *x* = −4, *y* = −8, *z* = −12; *t* = 3.25, *p* = 0.007 FWE-corrected, 26 voxels). There was a significant difference between controls and FEP patients (controls > FEP, maximal difference at *x* = 0, *y* = −16, z = −8; *t* = 4.63, *p* < 0.001 FWE-corrected, 39 voxels). Another way to follow-up the significant effect of group in the ANOVA to test our hypothesis of brain prediction-error signal following the pattern controls > at-risk patients > FEP patients, is by taking the prediction-error contrast value average for all 39 voxels that were significant in the ANOVA, and conducting planned paired group comparisons. This analysis also revealed that at-risk patients (mean = 0.66, SD = 54.86) were intermediate between the FEP patients and the controls, having a significantly smaller contrast value than controls: controls > at-risk patients, *p* = 0.034, one-tailed; but significantly greater than in FEP: at-risk > FEP patients, *p* = 0.001, one-tailed. The mean contrast value in the controls (mean = 24.44, SD = 50.12) was significantly greater than in FEP (mean = −55.03, SD = 50.70): controls > FEP, *p* < 0.001, one-tailed, confirming our hypothesis of controls > at-risk patient > FEP patients. In conclusion, these results show highest midbrain signalling in response to positive reward prediction error in controls, intermediate signalling in ARMS and lowest in FEP.

### Secondary region of interest prediction-error imaging results

No voxels passed our threshold on ANOVA in the associative-limbic striatum. There was a significant family-wise-error corrected group effect in the right DLPFC (maximal difference at *x* = 50, *y* = 26, *z* = 20; *t* = 3.53, *p* = 0.018 FWE-corrected, 20 voxels; Fig. 3). For each of these 20 significant voxels, family-wise error correcting for multiple comparisons, we performed planned two-group comparisons between using randomise to test our hypothesis of controls > at-risk patients > FEP patients. The results were not consistent with the hypothesis (Fig. 4). Although we found a significant difference between controls and FEP patients (controls > FEP, maximal difference at *x* = 50, *y* = 24, *z* = 20; *t* = 3.72, *p* < 0.001 FWE-corrected, 20 voxels), and between at-risk and FEP patients (at-risk > FEP, maximal difference at *x* = 50, *y* = 26, *z* = 20; *t* = 4.46, *p* < 0.001 FWE-corrected, 20 voxels), controls and at-risk patients did not differ significantly. We complemented the voxel based paired group comparisons with an analysis taking the prediction-error contrast value average for all 20 voxels in the DLPFC that were significant in the ANOVA, and conducting planned paired group comparisons to test our hypothesis of brain prediction-error signal following a pattern (controls > at-risk patients > FEP patients). This analysis was consistent with the voxel-wise paired group comparisons in that it did not support our hypothesis: the mean contrast values for these 20 voxels in controls (mean = 32.90, SD = 62.82) were significantly greater than in FEP (mean = −45.13, SD = 48.40): controls > FEP, *p* < 0.001, though not different from at-risk patients (mean = 36.29, SD = 53.03): controls > at-risk patients, *p* = 0.81. Mean contrast values in at-risk patients were significantly greater than in FEP (*p* < 0.001). These results show strong DLPFC activation in response to positive reward prediction error in controls, and nearly identical signalling in ARMS, but a significant deactivation in FEP.

As an explorational analysis, we analysed the left DLPFC, and we did not find any significant effect (*p* > 0.25).

### Symptom correlations

There were no significant correlations between midbrain or DLPFC activation total CAARMS symptom severity in FEP (midbrain rho = 0.03, *p* = 0.93; DLPFC rho = −0.27, *p* = 0.34) or the at-risk group (midbrain rho = 0.18, *p* = 0.33; DLPFC rho = 0.233, *p* = 0.21).

### Correlations with participants’ characteristics

No significant correlations between use of alcohol and performance, reaction times or brain signalling were detected.

## Discussion

We show evidence of significantly reduced midbrain signalling of reward prediction errors in patients with FEP and at-risk for psychosis. In addition, we also report DLPFC abnormalities in patients with FEP, showing that abnormalities associated with prediction-error processing in psychotic illness are not restricted to sub-cortical regions, consistent with previous results [12, 14, 51]. These results are not secondary to antipsychotic medication, because people with current or previous prescriptions of these drugs were excluded. Therefore, our findings extend previous findings reporting abnormal reward prediction-error signalling in the midbrain, striatum and cortex in a partly medicated sample of FEP patients (in which results held in a very small unmedicated subsample) [12], in medicated samples of schizophrenia patients [10, 11], and in the cortex and striatum of unmedicated, but not antipsychotic naive, samples of mixed FEP and chronic schizophrenia patients (mean age 27 years [13] or mean age 34 years [29]). We note that one previous study [25] tested an unmedicated, antipsychotic naive, schizophrenia sample and also reported midbrain alterations in a reward-associated task; this study, however, focussed on anticipation of reward and punishment rather than reward prediction error. To our knowledge, our study is, therefore, the first to document abnormal brain prediction-error signals in the dopaminergic midbrain with early-stage psychosis in an entirely antipsychotic naive sample.

Our study is also the first to examine reward prediction-error signalling in patients at-risk for developing psychosis, and as such it will require replication before definitive conclusions can be drawn, especially as there were only very small areas of difference in the at-risk patients. We found a mild abnormality in midbrain prediction-error signalling in the at-risk group that was intermediate in severity between psychotic illness and controls. Although the prediction-error signal in the dorsolateral prefrontal cortex was also disrupted in FEP patients, dorsolateral prefrontal cortex prediction-error signalling was relatively spared in the at-risk group, contrary to our hypothesis. These findings were similar in both the contrast-based prediction-error analysis presented in the main manuscript text, and in the alternative computationally informed approach presented in the Supplementary Material.

Conflicting evidence exists as to whether the same pathological mechanisms are responsible for both prodromal (sub-threshold) and severe psychotic symptoms [39]. Under the straightforward account of a dimensional theory of psychosis [52], the same pathology responsible for severe psychotic symptoms in schizophrenia should also be present (to a milder degree) in people with sub-threshold psychotic symptoms such as suspicions or mild hallucinations. Such an account would posit that people at-risk for psychosis due to the presence of sub-threshold psychotic symptoms would be characterised by a level of pathophysiological disruption that is of similar nature but lesser severity than the florid illness. The findings of the present study provide some support this theory for the dopaminergic midbrain, analogous to the pattern seen in a previous PET imaging study in the striatum [34]. However, we did not find significant associations with symptoms scores, which would be expected by a dimensional account. Another possibility, however, is that there may be qualitative differences in the pathology of sub-threshold and severe psychosis [53], which needs to be explored in future research. The currently still largely intact frontal prediction-error signalling in the at-risk group speculatively may be a mechanism that helps prevent a mild symptom becoming a severe one (e.g. a suspicion becoming a delusion), although longitudinal studies would be required to test such a mechanistic hypothesis.

Our study was not designed to be sensitive to prediction-error signalling in all cortical regions, and would be unlikely to be sensitive to auditory cortex predictive signals that have been implicated in the generation of auditory hallucinations [54, 55]. It would therefore be premature to conclude that all cortical prediction-error signalling is intact in at-risk patients. We note that two previous studies found preliminary evidence (not corrected for multiple comparisons) of enhanced frontal activation anticipating a reward in clinical risk groups using the monetary incentive delay paradigm [37, 38]. Although these prior findings may appear to contrast with our results of intact activation in the dorsolateral prefrontal cortex in the at-risk group, the studies are consistent in showing more prominent sub-cortical reductions in signalling in the at-risk state with no evidence of cortical reductions.

The probabilistic map of the dopaminergic midbrain, we used to assess midbrain activation [46], has been used in a number of prior studies [47, 48]. It combines the substantia nigra and the VTA; however, we did not seek to differentiate these structures. Differentiating activation amongst midbrain nuclei is generally challenging and confirmation of the precise anatomical abnormalities in psychosis could be facilitated with future developments in MRI technology such as the use of higher field strengths. We emphasise here that our results pertain to the region of the dopaminergic midbrain, acknowledging that the distinction between the substantia nigra and VTA is challenging in fMRI as well as that fMRI does not demonstrate the neurochemical origin of the signals observed. Our analysis of variance within a combined limbic and associative striatal region of interest did not demonstrate any voxels showing a significant group difference corrected for multiple comparisons. This could be due to a lack of power, with a small sample size in the FEP group and variable activation in the at-risk patients, or it could indicate areas of relatively spared function in some patients [32, 56, 57, 51]. The relatively small sample size, especially in the FEP group, is a limitation of the study, which is relevant for all regions of interest.

In our study, we did not detect any significant correlations between neuroimaging metrics and symptom scores. Symptom correlations often provide inconsistent results in schizophrenia research [58]; a key reason for this inconsistency is the difficulty of gathering a large enough sample, often requiring 50 or more patients for reasonable power, which is rare in single site neurobiology studies [58]. Therefore, our sample size is a clear limitation to assess symptom correlations. Although the focus of antipsychotic naive patients in the study is a strength in many ways, it is also a limitation, as the study sample is not completely representative of the entire population of first-episode psychosis. The combination of a requirement to have psychotic symptoms, and to undertake a cognitive task (albeit a simple one) in a scanner, but not be on antipsychotic medication, excludes some of the most severe patients who are simply to unwell to participate in this kind of research until their health improves on treatment.

In the analysis of choice performance and reaction time data we did not find any significant group differences. Win-stay/lose-shift analysis revealed that participants, independent of trial type (reward, bivalent or neutral), have a significantly higher probability to repeat the same response after a win than to shift after a loss, which is a clear indication for engagement and learning in a probabilistic learning task [59, 60]. The win stay probability was highest on reward trials, as expected, as these trials are the most predictable and consistently rewarding. However, also the win stay probability on bivalent trials shows that participants were engaging in the task and attempting to apply a learning strategy. The neutral trials show a lower probability rates and a less clear model learning behaviour. This, however, is not important for our analysis, as neutral trial imaging correlates are covariates of no interest. This analysis revealed subtle behavioural differences between the controls and the FEP patients: FEP patients have lower probabilities to repeat a response that lead to a win and a higher probability to shift after a loss, showing less stability in their decision making process.

An advantage of our study is that we use a paradigm that can generate assays of prediction-error signalling either by a traditional cognitive subtraction fMRI approach or by a computational modelling fMRI approach (Supplementary Material) as previously applied by others and ourselves [11, 12, 41, 59]. The convergent results indicate that the findings are not secondary to particular modelling strategy supplied, and are not confounded by outcome valence, which is often highly collinear with prediction error in many reinforcement learning studies, which has been raised as a concern raised by some researchers previously [43].

In conclusion, we document midbrain and cortical deactivations in prediction-error signals in antipsychotic naive FEP patients, supporting previous findings, and extending these to an antipsychotic naive sample. The findings in our at-risk group suggest a more nuanced account of the pathogenesis of symptoms. In the at-risk group, there was midbrain evidence of dysfunction intermediate between controls and FEP, but relatively spared DLPFC function in contrast to frank psychosis. Further investigations into areas of continuity and discontinuity between the at-risk and frank psychosis patients, including longitudinal designs, may bring insights into factors critical in the pathogenesis of psychotic illness.

## Electronic supplementary material

Supplementary Material
