# Supplementary material for: Abnormal reward prediction-error signalling in antipsychotic naive individuals with first-episode psychosis or clinical risk for psychosis
Source: Neuropsychopharmacology. 2018 Apr 5;43(8):1691–9. doi: 10.1038/s41386-018-0056-2 (PMC6006166; doi:10.1038/s41386-018-0056-2)
Supplement: Supplementary file 1 — Supplementary Material [file 41386_2018_56_MOESM1_ESM.docx]

Supplementary material

**Abnormal reward prediction error signalling in antipsychotic naïve individuals with first episode psychosis or clinical risk for psychosis**

Anna O Ermakova^1,2^*, Franziska Knolle^1,2^*, Azucena Justicia^1,3^, Edward T Bullmore^1,2,3^, Peter B Jones^1,2,3^, Trevor W Robbins^2,4^, Paul C Fletcher^1,2,3,5^, Graham K Murray^1,2,3^

1 Department of Psychiatry, University of Cambridge

2 Behavioural and Clinical Neuroscience Institute, University of Cambridge

3 Cambridgeshire and Peterborough NHS Foundation Trust

4 Department of Psychology, University of Cambridge

5 Institute of Metabolic Science, University of Cambridge

*joint first author with equal contribution to the work

Correspondence: Dr Graham K Murray, Department of Psychiatry, Box 189 Cambridge Biomedical Campus, Cambridge CB2 0QQ.

Email: gm285@cam.ac.uk***fMRI reward task (here described in greater detail than in main text)***

During the fMRI-scan, participants performed an instrumental discrimination learning task (Figure 1) involving monetary gains that required them to choose between two abstract visual stimuli (fractal pictures) displayed on a computer screen, to maximise pay-offs (Bernacer *et al*, 2013; Murray *et al*, 2008; Pessiglione *et al*, 2006; Seymour *et al*, 2007). On each trial, the participant chose one of the two stimuli, then feedback was provided. From the feedback, the participant learnt which of the pictures were more likely to give a reward of £1, or a loss of £1, and which ones were neutral. Each one of the three pairs of stimuli were presented in 30 trials (90 trials in total per subject). The stimuli within each pair led to a specific outcome with different probabilities: Reward trials: one picture led to a £1 win in 80% of trials and to neutral feedback in 20% of trials, the other picture led to a neutral outcome in 80% of trials and to a £1 win in 20% of trials; bivalent trials: there was a 50% chance of either losing or winning £1 (as in Seymour et al^1^); neutral trials: 80%/20% chance of receiving two kinds of neutral feedback (Figure 1, panel A). The order of the pictures presented and the position of the high-probability stimulus were counterbalanced across trials of the same valence and pseudo-randomised. To win money, the participants had to learn by trial and error, to learn which stimulus was more likely to produce a reward. The participants were informed that any money that they won during the experiment would be paid to them at the end of the study.

***fMRI data acquisition and analysis***

A Siemens Magnetom Trio Tim operating at 3T was used to collect imaging data. Gradient-echo echo planar T2*-weighted images depicting BOLD contrast were acquired from 35 non-contiguous oblique axial planes to minimise signal drop-out in the ventral regions. The repetition time (TR) was 1620ms, echo time (TE) was 30ms, flip angle was 65°, in-plane resolution was 3.0×3.0, matrix size was 64×64, field of view was 192×192 mm, and bandwidth was 2442 Hz/px. A total of 550 volumes per subject were acquired (35 slices each of 2 mm thickness, with an inter-slice gap of 1 mm) over one run. The first 5 volumes were discarded to allow for T1 equilibration effects. The acquisition time (TA) was 15.5min. The data were analysed using FSL software (FMRIB’s Software Library, www.fmrib.ox.ac.uk/fsl) version 5 (Jenkinson *et al*, 2012). Subjects’ data (i.e. first-level analysis) were processed using FMRI Expert Analysis Tool (FEAT). Functional images were realigned, motion corrected (MCFLIRT) and spatially smoothed with a Gaussian kernel 4 mm FWHM. A high-pass filter was applied (100s cut-off). All images were registered to the whole-brain echo-planar image (EPI) (i.e. functional image with the whole-brain field of view), then to the structural image of the corresponding subject (MPRAGE), and normalised to an MNI template. During the scanning, we recorded absolute (at-risk: 0.74 ±0.98; FEP: 0.87 ±0.93; controls: 0.59 ±0.42) and relative (at-risk: 0.12 ±0.04; FEP: 0.12 ±0.06; controls: 0.12 ±0.06) movement for the three groups. There were no significant differences between groups (absolute: p=0.47; relative: p=0.98).

***FEP diagnoses***

8 patients met ICD10 criteria for schizophrenia (F20), 2 for schizoaffective disorder (F25), 1 for severe depressive episode with psychotic symptoms (F32.3), and 3 for psychosis not otherwise specified (F29). In Supplementary Figure 4, we provide the analysis excluding the one FEP patient with a severe depressive episode. The results are identical to the original findings.

***Effect of antidepressants on behavioural performance and imaging results***

Antidepressant medication was taken by one control (sertraline), four FEP (two fluoxetine and two sertraline), and eight ARMS (two fluoxetine, two citalopram, one sertraline, one amitriptyline (low dose), two unknown). The use of antidepressants differed significantly between controls and patients (p=0.007). The use of antidepressants did not appear to impact the performance across all groups (all p>0.1). When conducting T-tests within each group, we found that there were no differences between FEP or controls on antidepressants compared to those not on antidepressants, neither in the dopaminergic midbrain nor in the DLPFC. In the ARMS patients, we found significant differences between those on antidepressants and those not on antidepressants (on: mean=44.14 (±50.29); off: mean=-15.15 (±48.29); F(28)=2.94, p=0.006). ARMS patients who were taking antidepressants showed a more normalised signal similar to controls (p=0.3). Those ARMS patients who were not taking antidepressant showed a dopaminergice midbrain signal intermediate between FEP (p=0.029) and controls (p=0.004), confirming the original results.

| **Supplementary Table 1.** Group probablilities for win-stay/lose-shift behaviour. | | | | |
| --- | --- | --- | --- | --- |
| Groups | Stay-switch behaviour | Trial type | Mean | SE |
| Controls | Win-stay | Reward | 84.35 | 3.33 |
|  |  | Bivalent | 74.77 | 3.08 |
|  |  | Neutral | 62.17 | 3.54 |
|  | Lose-shift | Reward | 23.94 | 3.59 |
|  |  | Bivalent | 47.70 | 2.95 |
|  |  | Neutral | 39.16 | 3.13 |
| At-risk | Win-stay | Reward | 81.44 | 3.79 |
|  |  | Bivalent | 68.05 | 3.51 |
|  |  | Neutral | 62.65 | 4.03 |
|  | Lose-shift | Reward | 28.83 | 4.09 |
|  |  | Bivalent | 46.19 | 3.37 |
|  |  | Neutral | 47.48 | 3.57 |
| FEP | Win-stay | Reward | 74.77 | 5.55 |
|  |  | Bivalent | 63.44 | 5.13 |
|  |  | Neutral | 44.50 | 5.90 |
|  | Lose-shift | Reward | 48.61 | 5.99 |
|  |  | Bivalent | 50.95 | 4.93 |
|  |  | Neutral | 60.36 | 5.23 |


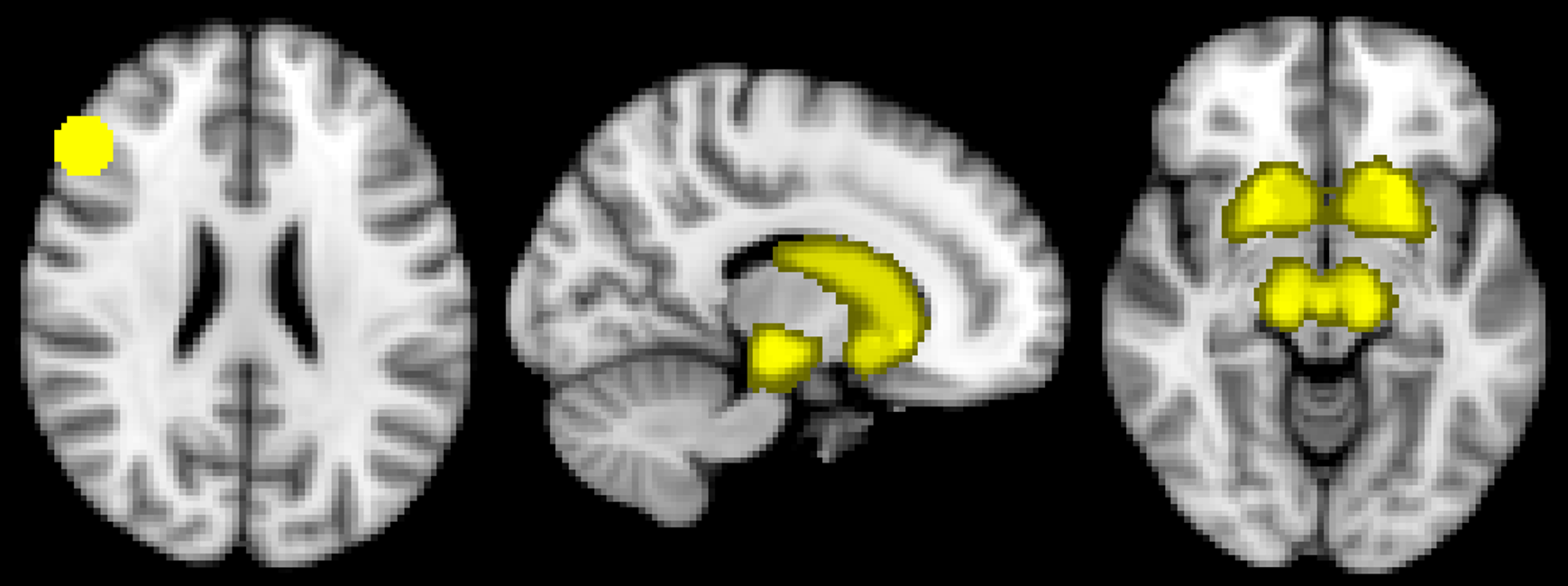


**Supplementary Figure 1.** 3 anatomical masks of regions of interest used for analysis. Areas marked in yellow show anatomical masks. (DLPFC: 515 voxels, extracted utilising a sphere, 10 mm, centred at x=50, y30=, z=28, size: 4.1cm^3^, based on our previous work (Corlett *et al*, 2007); associative and limbic striatum , including caudate head and nucleus accumbens: 1800 voxels, size: 14.4cm^3^, extracted using a hand drawn mask based on operational criteria (Mawlawi *et al*, 2001); dopaminergic midbrain (combining VTA and substantia nigra): 136 voxels, size: 1.1cm^3^, provided Murty and colleagues (Murty *et al*, 2014). Voxel size was 2mm x 2mm x 2mm.


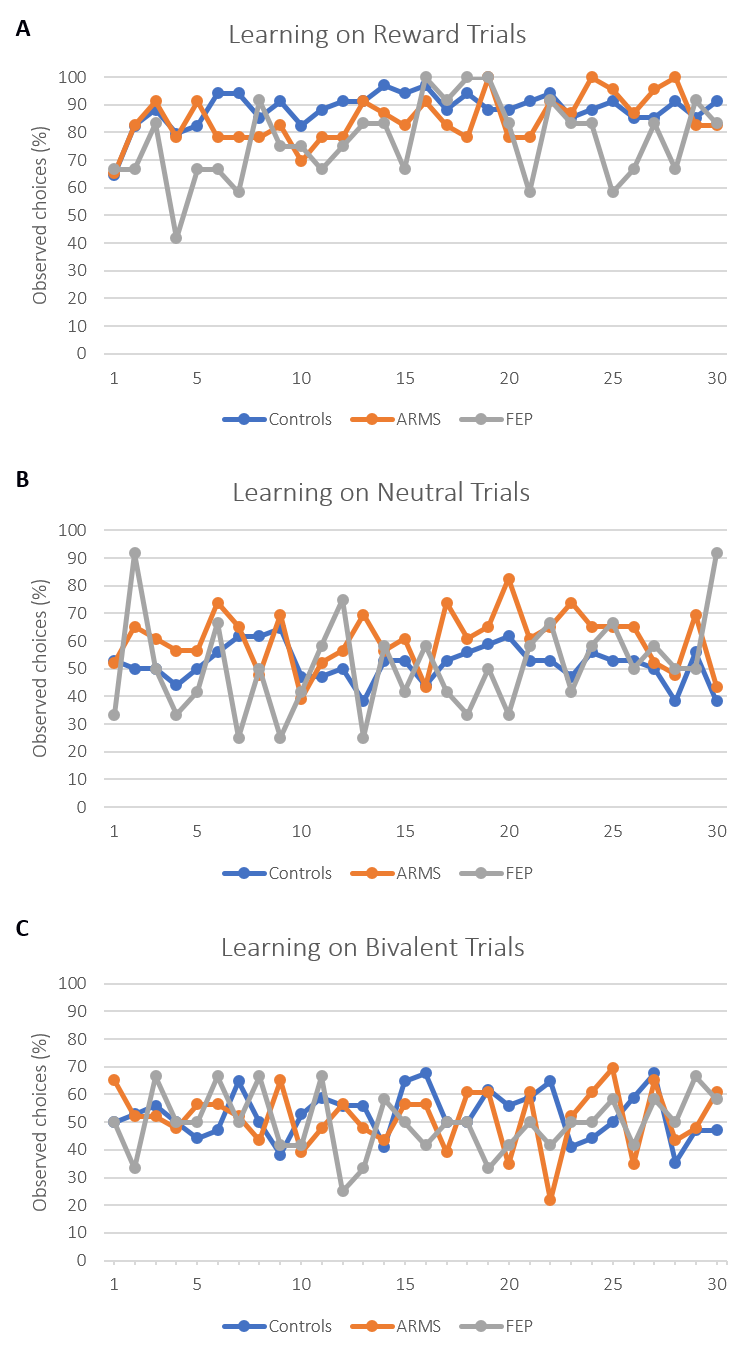


**Supplementary Figure 2.** Learning curves show a trial-by-trial depiction of observed correct “choices” per group (controls, at-risk (ARMS), first episode psychosis (FEP)).


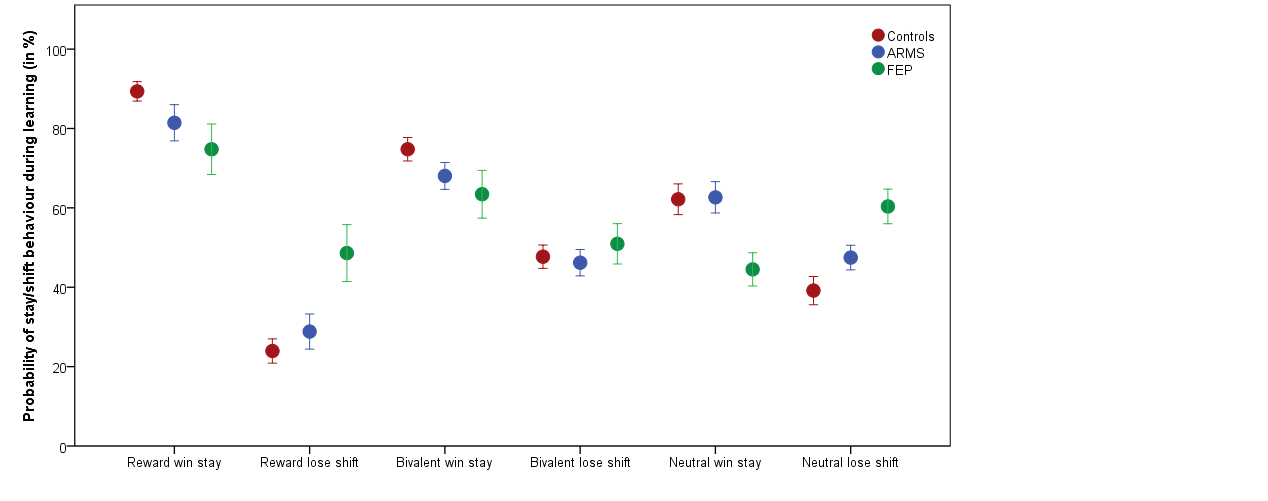


**Supplementary Figure 3.** Probabilities of win-stay and lose-shift behaviour across groups and trial types. All participants are more likely to repeat the same response after a win.


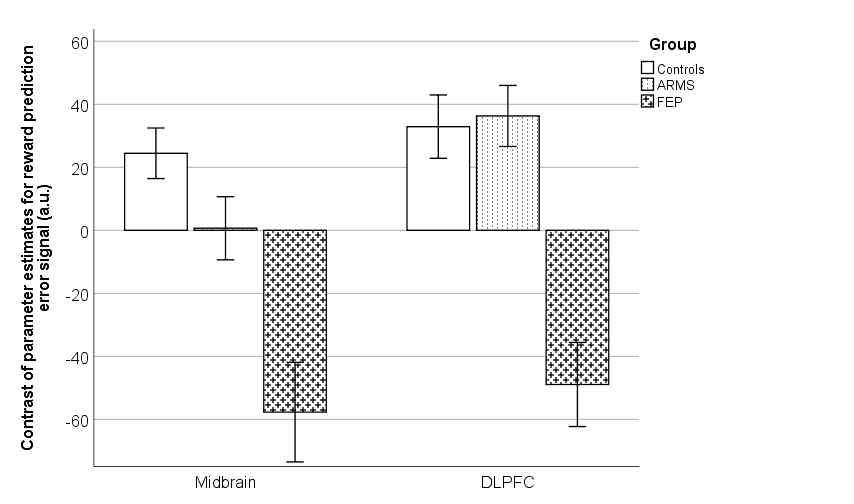


**Supplementary Figure 4.** Bar chart shows the mean prediction error contrast values according to group excluding one FEP patient with a severe depressive episode. The results are identical to our original findings. Controls and ARMS are significantly different from FEP patients in both regions (Con>FEP: dopaminergic midbrain: t=4.95; p<0.001; DLPFC: t=4.29, p<0.001; ARMS>FEP: dopaminergic midbrain: t=3.17; p=0.003; DLPFC: t=4.97, p<0.001). Error bars show ±1 SE; a.u. is arbitrary units.


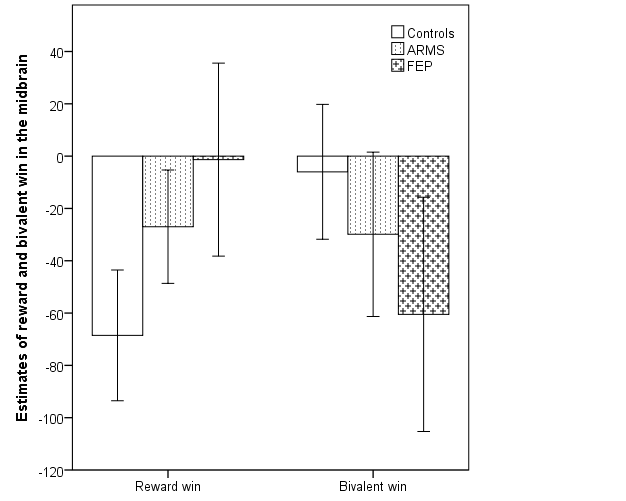


**Supplementary Figure 5**. Parameter estimates at 0, -16, -8 (left midbrain). The bar graph shows the midbrain parameter estimates for the at-risk patients (ARMS), FEP and control group in response to wins on reward and bivalent trials. The differing effect observed between FEP and controls on the prediction error contrast seems to be driven by a combination of patients’ reduced activation to wins on bivalent trials compared to controls and enhanced activation to reward trial wins compared to controls. Error bars represent standard error of the mean.


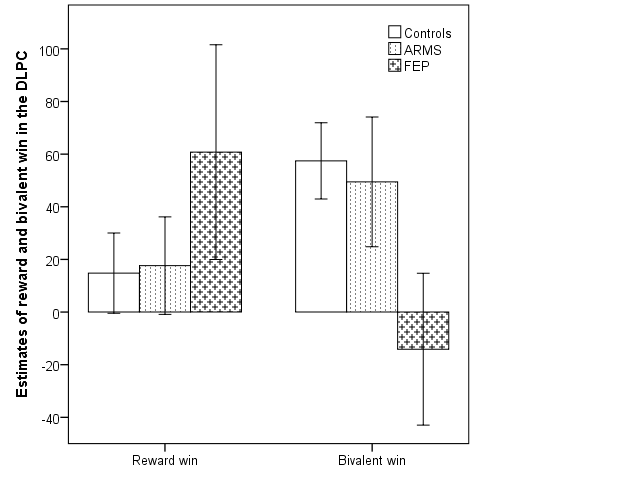


**Supplementary Figure 6**. DLPFC, parameter estimates at 48, 34, 22. The bar graph shows activations in the DLPFC for the at-risk patients (ARMS), FEP and control group to reward and bivalent trials. The differing effect in the group analysis seems to be driven by the inverted pattern of activation in response to reward and bivalent trials in the FEPs compared to the at-risk and control group. Error bars represent standard error of the mean.

***Computational model***

Participants who scored under 50% on the reward trials were excluded from the analysis of the computational model, as this may reflect non-optimal learning (this occurs for example, when participants learn that the low likelihood stimulus predicts a reward reliably). We excluded two FEP patients leaving 12, seven at-risk patients leaving 23, and five controls leaving 34.

Using a standard reinforcement learning algorithm, we modelled the subject’s choices on a trial basis. As previously (Bernacer *et al*, 2013; Murray *et al*, 2008) we applied a basic Q learning algorithm. The model estimates the expected reward (Q-value) for picking either A (Qa) or B (Qb) in a given stimulus pair taking the subjects’ previous choices and outcomes into account (α = learning rate). Prior to learning (i.e. at the beginning of a task) Q is set to zero, which is then updated according to the following rule, for picking A:

$$Qa(t+1)=Qa(t)+\alpha*\delta(t)$$

The prediction error was defined as

$$\delta(t=R(t)-Qa(t)$$

with R(t) being the reinforcement through the actual outcome for choosing A at trial t. Therefore, the prediction error δ(t) is the difference the actual (R(t)) and expected (Qa(t)) outcome. The reinforcement values were +1 for winning, 0 for neutral, and -1 for loosing outcomes.

Applying a standard stochastic decision rule (i.e. SoftMax rule) we then calculated the probabilities for choosing A or B according to the values Qa and Qb (example for A):

$$\mathrm{Pa}\left( t \right)=\frac{e^{\frac{\mathrm{Qa}\left( t \right)}{\beta}}}{e^{\frac{\mathrm{Qa}\left( t \right)}{\beta}}+e^{\frac{\mathrm{Qb}\left( t \right)}{\beta}}}$$

The constants α and β were calculated to present the best model fit (i.e. maximized probability or likelihood of actual choice) for all participants individually.

To calculate log-likelihood, we followed the method of Pessiglione et al (2006)^3^. For a given value of alpha and beta, we calculate the probability of the observed choice for each subject for each trial. The probability of the series of choices made by participant A on reward trials is the product of the probabilities of the individual trials. The log of the probability of the series of choices on reward trials made by participant A is equal to the of the sum of the log-probabilities. The maximal log probability indicated the optimal model parameters for each individual. When we estimated α and β for each participant individually, we did not find significant differences between groups (α: F=0.85, df=2 p=0.43, β: F=2.73, df=2 p=0.29).

For the learning model used for the imaging analysis, we generated an average set of parameters per trial type across all participants. We detected α=0.44 and β=0.43 for reward trials and α=0.34 and β=1.16 for bivalent trials to provide the best fit for the data (Supplementary Figures 7-10 and Table 1). There was a significant difference of goodness of fit (log-likelihood) of the computational model for the behavioural choices with the model fitting better for reward trials (F=68.68, df=1, p<0.001), but no differences between groups (F=2.39, df=2 p=0.1). The learning model was then used to generate statistical regressors to be used in the imaging analysis of the prediction error.

***fMRI data analysis (based on computional modelling)***

In support of results as presented in the main manuscript, we also ran an additional analysis with a general linear model where prediction error regressors were determined by a computational Q-learning model as we and others have used previously (Bernacer *et al*, 2013; Murray *et al*, 2008; Pessiglione *et al*, 2006). In this analysis, we analyse a slightly reduced sample, based on performance criteria as described above. The results are comparable and show that the results are not dependent on a particular approach.

We used a general statistical linear model for our analysis. Based on our learning model, we created the following seven regressors: 1) onset of the bivalent cues; 2) onset of the neutral cues; 3) onset of the reward cues; 4) neutral outcome onsets (neutral feedback) during both neutral and reward trials; 5) winning outcome in the reward and bivalent trials; 6) positive prediction error in the reward and bivalent trials at the time of reward feedback. We orthogonalised the positive predictions error regressor (6) with respect to the outcome regressor (5) to detect BOLD activation uniquely explained by the prediction error (Erdeniz *et al*, 2013).

All regressors were modelled as 2 s events and convolved with a canonical double-gamma response function. We added temporal derivatives to the model to take into account possible variation in the haemodynamic response function and we included motion parameters. Our contrast of interest corresponded to activation associated with a combined positive prediction error during reward feedback on reward and bivalent trials (i.e. our Contrast of Parameter Estimates, termed COPE in FSL, was formed by a +1 contrast on regressor 6).

For the group analysis (i.e. higher-level analysis) the prediction error contrast was the outcome variable and group was the predictor. We used permutation based statistics using the FSL tool randomise, utilising threshold-free-cluster enhancement, which enhances cluster-like structures but remains fundamentally a voxel-wise statistical testing method (Winkler *et al*, 2014). We used the variance smoothing option (3mm) as recommended for experiments with modest sample sizes (Nichols and Holmes, 2002). We had one primary region of interest: the dopaminergic midbrain (using the probabilistic atlas of ­­­­­­Murty and colleagues (Murty *et al*, 2014), combining the VTA and the substantia nigra); and two secondary regions of interest, the first, a single region composed of the associative and limbic striatum (using a hand drawn mask based on operational criteria (Martinez *et al*, 2003; Mawlawi *et al*, 2001)), and the other, the right dorsolateral frontal cortex (using a sphere, 10mm, centred at x=50, y=30, z=28, based on our previous work (Corlett *et al*, 2007)) (see Supplementary Figure 1). We conducted ANOVA across groups within these regions of interest using randomise. The significance threshold was set at alpha of 0.05, family-wise error corrected for multiple comparisons. In order to visualize the differences between the three groups, and to test the hypothesis of controls > at risk patients > FEP patients, we conducted planned paired group tests on voxels that were significant in the ANOVA.

***Modelled prediction error imaging results: ANOVA across three groups, whole brain***

On whole brain analysis, there were no group differences that passed our statistical threshold corrected for multiple comparisons.

***Modelled prediction error imaging: ANOVA across three groups in primary region of interest***

We conducted an ANOVA using FSL randomise, using our modelled prediction error contrast of interest as the outcome variable and group as the predictor variable in the primary region of interest, the dopaminergic midbrain (maximal difference at x=-8, y=-10, z=-8; t=3.17, p=0.049 FWE corrected, 2 voxels).

On the significant voxels, we then performed a planned comparison between each pair of groups using randomise to test our hypothesis of controls > at risk patients > FEP patients. The results were consisrent with the hypothesis: at-risk patients significantly differed from FEP patients and controls (controls>at-risk patients maximal difference at x=-6, y=-12, z=-8; t=2.51, p=0.013 FWE corrected; at-risk>FEP patients, maximal difference at x=-8, y=-10, z=-8; t=2.64, p=0.013 FWE corrected). There was also a significant difference between controls and FEP patients (controls>FEP, maximal difference at x=-6, y=-12, z=-8; t=3.75, p<0.001 FWE corrected). To complement this analysis, we examined a slightly larger region, by incuding the voxels (n=7) that showed a group difference on ANOVA were significant at p<0.1 FWE corrected, and repeated paired group comparisons. We found a significant difference between controls and FEP patients (controls>FEP, maximal difference at x=-4, y=-12, z=-8; t=3.39, p<0.001 FWE corrected, 7 voxels). At-risk patients significantly differed from FEP patients and controls (controls>at-risk patients maximal difference at x=-6, y=-12, z=-8; t=2.42, p=0.032 FWE corrected, 6 voxels; at-risk>FEP patients, maximal difference at x=-10, y=-10, z=-8; t=2.55, p=0.025 FWE corrected, 7 voxels).

***Modelled prediction error imaging: ANOVA across three groups in secondary regions of interest***

We conducted an ANOVA using FSL randomise, using our modelled prediction error contrast of nterrst as the outcome variable and group as the predictor variable in the secondary regions of interest, the striatal ROI and the DLPFC ROI. We did not find significant differences in the striatal ROI. There was a significant family-wise-error corrected group difference on ANOVA in the DLPFC (maximal difference at x=52, y=24, z=30; t=3.33, p=0.05 FWE corrected, 1 voxel). On this peak voxel, we then performed a planned comparison between each pair of groups using randomise. We found a significant difference between controls and FEP patients (controls>FEP, maximal difference at x=52, y=24, z=30; t=3.92, p<0.001). There was a significant difference between at-risk and FEP patients (at-risk>FEP, maximal difference at x=52, y=24, z=30; t=2.56, p=0.008). However, controls and at-risk patients did not differ. To complement this analysis, we examined a slightly larger region, by incuding the voxels (n=20) that showed a group difference on ANOVA were significant at p<0.1 FWE corrected, and conducted paired group comparisons (Supplementary Figure 13). We found a significant difference between controls and FEP patients (controls>FEP, maximal difference at x=46, y=22, z=26; t=3.56, p<0.001 FWE corrected, 20 voxels). We also found a significant difference between at-risk and FEP patients (at-risk>FEP, maximal difference at x=50, y=24, z=20; t=3.28, p=0.01 FWE corrected, 18 voxels). Controls and at-risk patients did not differ.

| **Supplementary Table 3. List of individual modelling parameters** | | | | | | |
| --- | --- | --- | --- | --- | --- | --- |
| at-risk | Alpha Reward | Beta Reward | LL Reward | Alpha Bivalent | Beta Bivalent | LL bivalent |
| 1 | 0.37 | 0.25 | -6.0534 | 0.08 | 0.05 | -8.7634 |
| 2 | 0.06 | 0.05 | -4.7197 | 0.1 | 0.2 | -17.416 |
| 3 | 0.45 | 0.05 | -0.6939 | 0.07 | 0.05 | -9.3805 |
| 4 | 0.03 | 0.05 | -5.4143 | 0.45 | 1.65 | -20.41 |
| 5 | 1 | 1.6 | -19.187 | 0.01 | 5 | -20.804 |
| 6 | 1 | 0.4 | -15.923 | 0.63 | 1.4 | -19.6 |
| 7 | 0.39 | 0.25 | -5.6992 | 0.23 | 0.2 | -12.119 |
| 8 | 0.01 | 5 | -20.803 | 0.42 | 2.05 | -20.569 |
| 9 | 0.01 | 5 | -20.812 | 0.34 | 2.4 | -20.471 |
| 10 | 0.03 | 0.1 | -12.032 | 0.27 | 0.55 | -18.36 |
| 11 | 0.08 | 0.1 | -4.0311 | 0.02 | 0.05 | -12.943 |
| 12 | 0.11 | 0.55 | -18.37 | 0.03 | 0.05 | -16.818 |
| 13 | 0.38 | 0.25 | -5.9283 | 0.1 | 0.55 | -19.103 |
| 14 | 0.49 | 0.05 | -1.3933 | 0.04 | 0.05 | -13.873 |
| 15 | 0.49 | 0.05 | -1.3933 | 0.01 | 0.05 | -20.057 |
| 16 | 0.45 | 0.05 | -0.6939 | 0.04 | 0.05 | -17.437 |
| 17 | 0.95 | 0.05 | -4.8997 | 1 | 1.3 | -17.119 |
| 18 | 0.49 | 0.05 | -1.3933 | 0.02 | 0.05 | -17.916 |
| 19 | 0.18 | 0.05 | -3.6895 | 0.04 | 0.05 | -16.835 |
| 20 | 0.17 | 0.05 | -7.3268 | 0.01 | 5 | -19.459 |
| 21 | 1 | 2.95 | -19.934 | 0.05 | 0.1 | -19.86 |
| 22 | 0.15 | 0.25 | -10.036 | 0.01 | 5 | -20.797 |
| 23 | 0.93 | 0.35 | -10.245 | 0.44 | 0.65 | -16.883 |
| 24 | 0.95 | 0.05 | -5.5928 | 0.92 | 0.65 | -13.545 |
| 25 | 0.45 | 0.05 | -0.6939 | 0.04 | 0.05 | -14.631 |
| 26 | 0.01 | 5 | -20.818 | 0.13 | 5 | -20.794 |
| 27 | 0.05 | 0.25 | -16.779 | 0.89 | 5 | -20.705 |
| 28 | 0.6 | 5 | -20.072 | 0.44 | 0.75 | -17.463 |
| 29 | 0.03 | 0.1 | -11.177 | 0.01 | 0.05 | -20.563 |
| 30 | 0.01 | 0.25 | -20.677 | 1 | 2.05 | -18.564 |
| FEP |  |  |  |  |  |  |
| 1 | 0.5 | 0.2 | -7.747 | 0.06 | 0.05 | -7.0162 |
| 2 | 0.33 | 0.55 | -18.181 | 0.91 | 1.55 | -18.26 |
| 3 | 0.2 | 1.2 | -20.069 | 0.01 | 5 | -20.797 |
| 4 | 0.01 | 5 | -20.836 | 0.06 | 0.05 | -7.0934 |
| 5 | 0.83 | 0.1 | -3.3697 | 0.1 | 5 | -20.784 |
| 6 | 0.45 | 0.05 | -0.6939 | 1 | 4.85 | -20.437 |
| 7 | 0.01 | 5 | -18.034 | 0.01 | 0.2 | -18.631 |
| 8 | 0.4 | 0.3 | -8.74 | 1 | 1.85 | -18.176 |
| 9 | 0.83 | 0.75 | -17.74 | 0.56 | 0.7 | -16.296 |
| 10 | 1 | 0.55 | -14.852 | 0.02 | 0.05 | -17.914 |
| 11 | 0.91 | 0.1 | -4.8747 | 0.46 | 1.15 | -18.773 |
| 12 | 1 | 0.9 | -18.178 | 0.86 | 0.85 | -15.182 |
| 13 | 1 | 0.9 | -18.702 | 0.02 | 0.05 | -19.992 |
| 14 | 0.18 | 0.85 | -19.097 | 0.01 | 5 | -20.82 |
| Controls |  |  |  |  |  |  |
| 1 | 0.04 | 0.05 | -4.3052 | 1 | 3.05 | -17.299 |
| 2 | 0.35 | 0.25 | -7.7495 | 0.01 | 0.05 | -20.48 |
| 3 | 0.01 | 0.05 | -14.202 | 0.04 | 0.1 | -18.147 |
| 4 | 0.92 | 0.05 | -4.1126 | 0.07 | 0.05 | -11.029 |
| 5 | 0.45 | 0.05 | -0.6939 | 0.03 | 0.05 | -17.53 |
| 6 | 0.92 | 0.05 | -3.3978 | 0.07 | 0.05 | -7.6752 |
| 7 | 0.49 | 0.05 | -1.3933 | 0.17 | 0.35 | -19.491 |
| 8 | 0.83 | 0.1 | -3.3697 | 0.14 | 5 | -20.789 |
| 9 | 0.45 | 0.05 | -0.6939 | 0.5 | 1.45 | -19.471 |
| 10 | 0.03 | 0.05 | -5.9377 | 0.03 | 0.1 | -17.367 |
| 11 | 0.49 | 0.05 | -1.3933 | 0.52 | 0.85 | -17.935 |
| 12 | 0.03 | 0.1 | -8.3251 | 0.14 | 0.3 | -18.793 |
| 13 | 0.45 | 0.05 | -0.6939 | 0.16 | 0.25 | -17.438 |
| 14 | 0.45 | 0.05 | -0.6939 | 0.12 | 0.1 | -11.941 |
| 15 | 0.03 | 0.05 | -4.0278 | 0.38 | 0.8 | -17.856 |
| 16 | 0.83 | 0.1 | -3.3697 | 0.05 | 0.05 | -15.4 |
| 17 | 0.83 | 0.1 | -3.3697 | 0.79 | 0.8 | -15.53 |
| 18 | 0.45 | 0.05 | -0.6939 | 0.41 | 0.7 | -16.851 |
| 19 | 0.01 | 5 | -20.827 | 1 | 3.05 | -19.902 |
| 20 | 0.59 | 0.95 | -18.69 | 0.72 | 1 | -16.787 |
| 21 | 0.49 | 0.05 | -1.3933 | 0.03 | 0.05 | -16.304 |
| 22 | 0.02 | 0.05 | -9.4716 | 0.37 | 0.25 | -9.2947 |
| 23 | 0.45 | 0.05 | -0.6939 | 0.02 | 0.05 | -19.44 |
| 24 | 0.06 | 0.05 | -4.7196 | 0.01 | 5 | -15.263 |
| 25 | 0.64 | 2.05 | -20.447 | 0.25 | 2.15 | -20.49 |
| 26 | 0.04 | 0.05 | -5.2654 | 1 | 1.05 | -16.663 |
| 27 | 0.02 | 0.05 | -19.141 | 0.99 | 0.15 | -10.382 |
| 28 | 0.05 | 0.1 | -5.3971 | 0.92 | 0.05 | -5.8118 |
| 29 | 0.49 | 0.05 | -1.3933 | 0.73 | 0.05 | -2.7491 |
| 30 | 0.2 | 2.55 | -19.983 | 1 | 1.75 | -18.459 |
| 31 | 1 | 0.55 | -14.06 | 0.45 | 1.45 | -18.725 |
| 32 | 1 | 5 | -20.694 | 0.59 | 0.6 | -14.307 |
| 33 | 0.5 | 0.4 | -13.323 | 0.12 | 0.2 | -18.628 |
| 34 | 0.01 | 0.05 | -20.102 | 0.49 | 1.1 | -19.405 |
| 35 | 0.04 | 0.05 | -15.021 | 0.04 | 0.05 | -17.547 |
| 36 | 0.49 | 0.05 | -1.3933 | 1 | 2.35 | -18.765 |
| 37 | 0.45 | 0.05 | -0.6939 | 0.25 | 0.3 | -13.992 |
| 38 | 0.49 | 0.05 | -1.3933 | 0.05 | 0.1 | -16.095 |
| 39 | 0.01 | 5 | -20.828 | 1 | 0.05 | -10.397 |


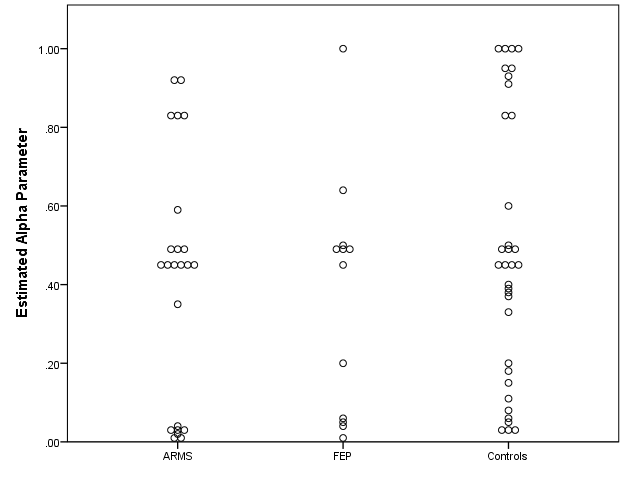


**Supplementary Figure 7.** Estimates of learning rate (α parameter) for reward trials.


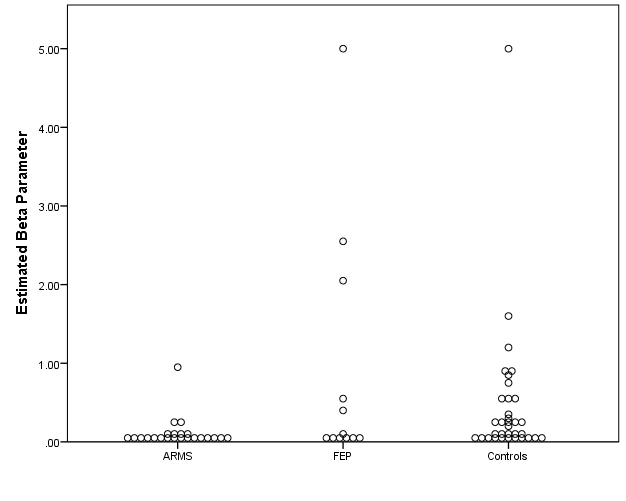


**Supplementary Figure 8.** Estimates of learning rate (α parameter) for bivalent trials.


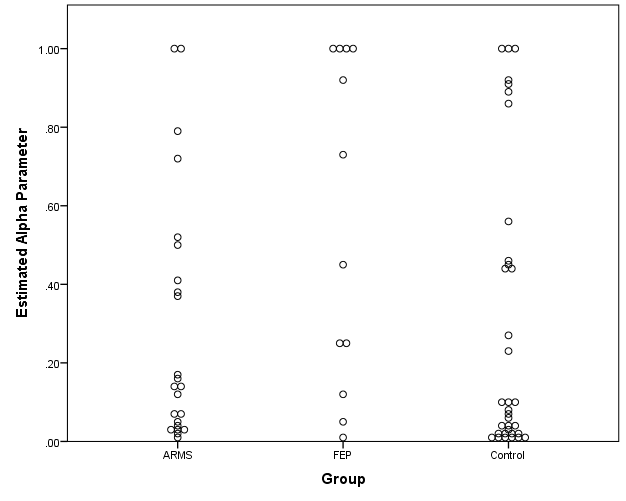


**Supplementary Figure 9.** Estimates of exploration-exploitation β parameter for reward trials.


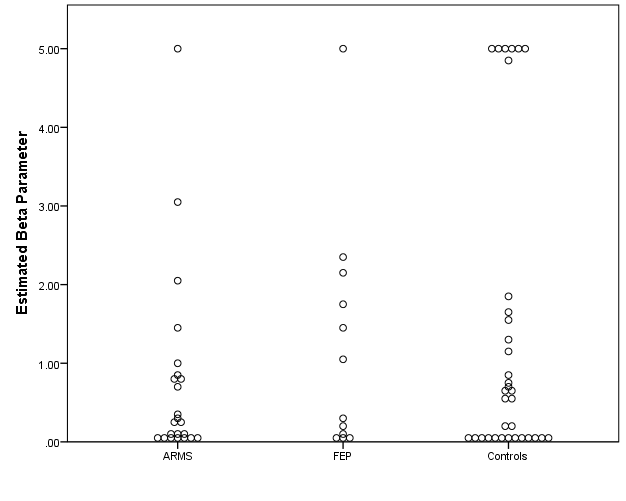


**Supplementary Figure 10.** Estimates of exploration-exploitation β parameter for bivalent trials.


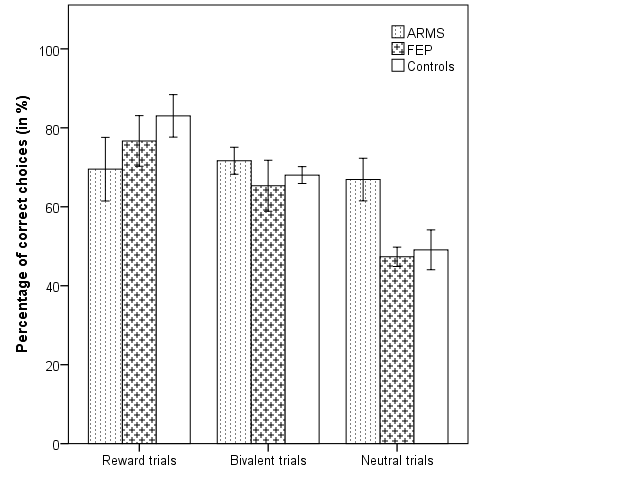


**Supplementary Figure 11.** Behavioural results: Percentage of “correct” (high-likelihood) choices stratified by trial type and participant group. On bivalent and neutral trials, “correct” stimulus is arbitrarily defined. Within each group, participants chose the “correct” stimulus on reward trials more frequently than on other trial types. Error bars are ±1 SE.

**
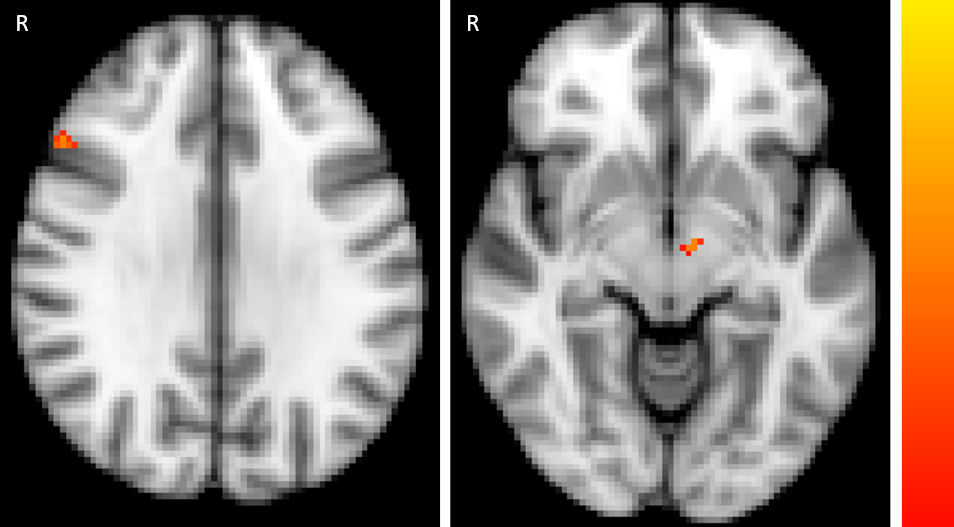
**

**Supplementary Figure 12.** Group differences on region of interest analysis of reward prediction error signals in the dopaminergic midbrain (right panel, z=-8) and dorslolateral prefrontal cortex (left panel, z=30). Peak voxels were significant at p<0.05 family wise error corrected; for illustrative purposes results the results are shown here thresholded at p<0.1 family-wise error corrected. Colour bar depicts corrected voxel p-value from 0.001 (yellow) to 0.1 (red).


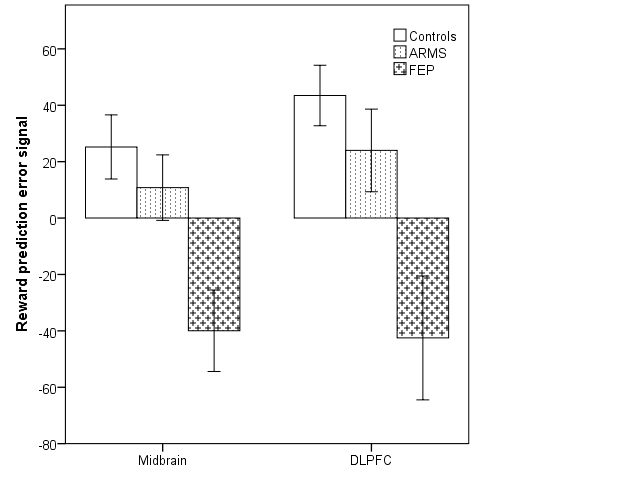
**Supplementary Figure 13.** Bar chart showing mean reward prediction error contrast values (Constrast of Parameter Estimates, or COPEs) extracted from voxels differentiating the groups shown in Supplementary Figure 12). Error bars represent ±1 SE. At-risk patients (ARMS) show intermediate values in the dopaminergic midbrain compared to controls and first episode psychosis patients (FEP). FEP patients show reduced values in the midbrain and the prefrontal cortex.


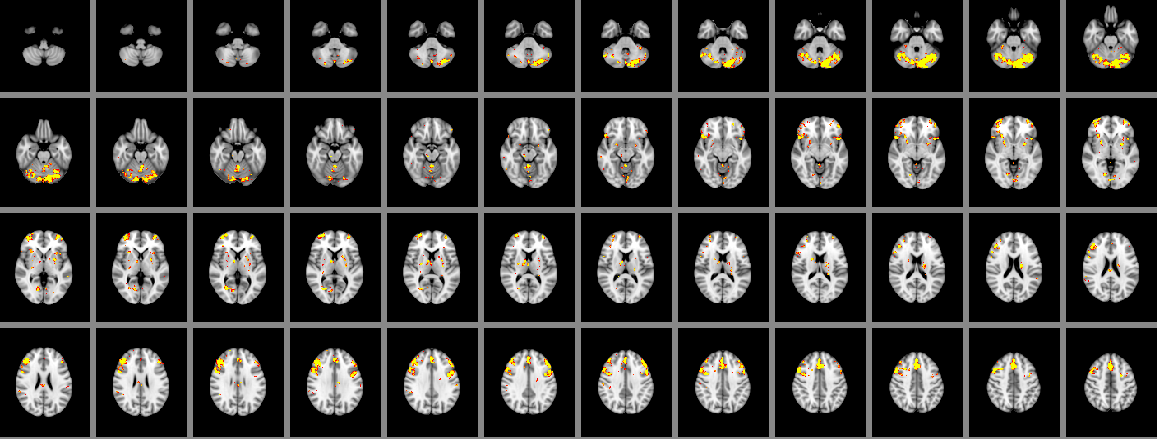


**Supplementary Figure 14.** Contrast Based Analysis: Prediction Error Results (Reward win – Bivalent win) in the whole sample, thresholded at z=2.5 (red) uncorrected. Yellow indicates z>3.

| **Supplementary Table 4: Active clusters of the contrast based analysis for Reward win minus Bivalent win in the whole sample, thresholded at z=2.5 uncorrected, cluster size greater than 5 voxels.** | | | | | | |
| --- | --- | --- | --- | --- | --- | --- |
| Anatomical structure | Hemisphere | Cluster size (voxels) | Peak Z-score | MNI Coordinates | | |
|  |  |  |  | x | y | z |
| Cerebellum | L/R | 2909 | 5.55 | -12 | -76 | -28 |
| Lobus I-IV | L/R | 246 | 4.47 | 0 | -50 | -14 |
| Lobus IV/V | L | 28 | 3.8 | -34 | -42 | -36 |
|  | R | 20 | 3.04 | 28 | -36 | -28 |
| Crus II | R | 20 | 3.47 | 24 | -80 | -42 |
| DLPFC | R | 1275 | 4.63 | 46 | 32 | 30 |
|  | L | 469 | 4.03 | -50 | 22 | 34 |
| ACC | L/R | 691 | 4.94 | 4 | 26 | 44 |
| PCC | L/R | 41 | 3.13 | 0 | -22 | 28 |
| Thalamus | R | 109 | 3.72 | 10 | -2 | 8 |
|  | L | 15 | 3.25 | -20 | -24 | 14 |
| Putamen | R | 92 | 3.66 | 24 | 6 | -2 |
|  | R | 19 | 3.3 | 26 | -8 | 8 |
|  | R | 6 | 2.84 | 28 | 0 | 0 |
|  | L | 112 | 3.92 | -26 | 2 | -2 |
|  | L | 24 | 3.21 | -26 | -14 | 8 |
| Caudate | R | 13 | 3.3 | 20 | 2 | 16 |
|  | L | 63 | 3.28 | -18 | -12 | 20 |
| Substantian Nigra/VTA | R | 17 | 3.16 | 10 | -26 | -14 |
| IFG | R | 248 | 4.49 | 50 | 18 | -6 |
|  | L | 136 | 3.78 | -46 | 16 | -8 |
| Intracalcarine Cortex | R | 176 | 3.79 | 22 | -68 | 6 |
|  | L | 16 | 3.28 | 30 | -18 | 0 |
|  | L | 17 | 3.08 | -8 | -74 | -4 |
| MFG | R | 21 | 2.98 | -38 | 2 | 44 |
|  | L | 17 | 3.4 | -26 | 0 | 46 |
| Frontal Lobe | R | 377 | 4.28 | 38 | 60 | 6 |
|  | R | 35 | 3.4 | 32 | 46 | 16 |
|  | L | 230 | 4.27 | -34 | 58 | 0 |
|  | L | 61 | 3.55 | -44 | 52 | -2 |


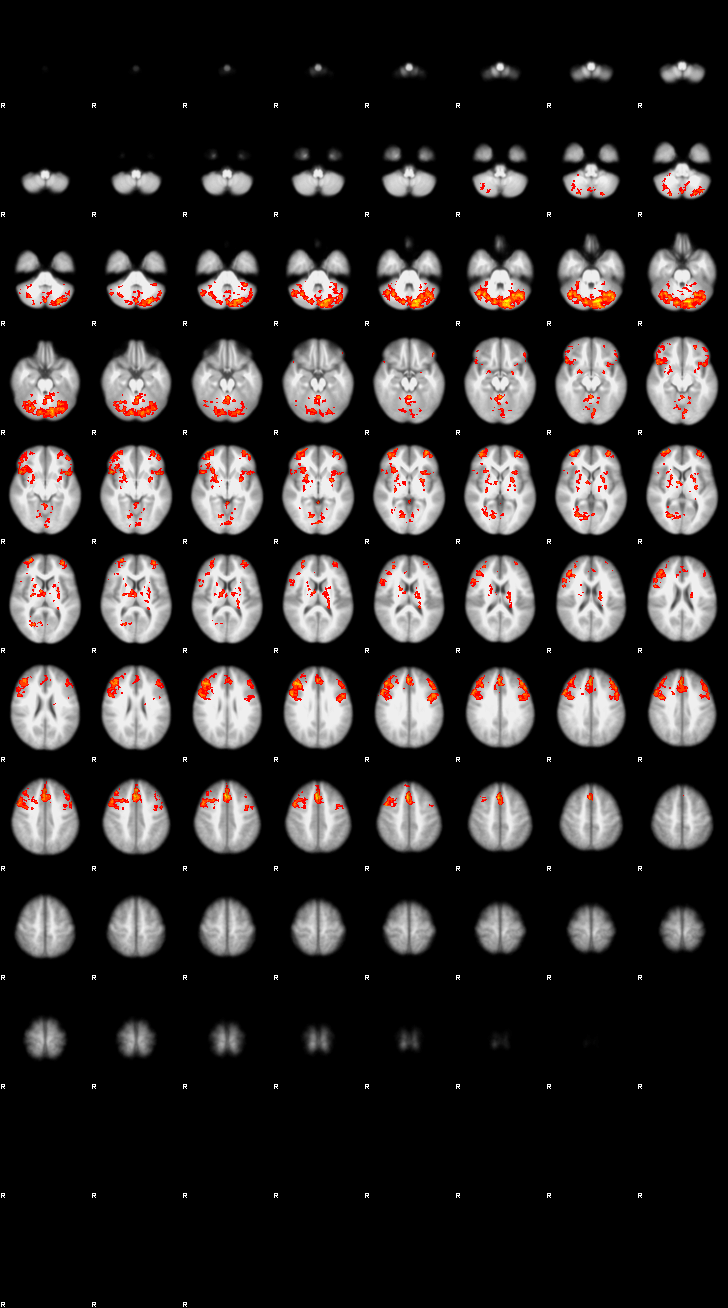


**Supplementary Figure 15.** Whole brain analysis across all participants pooled. Prediction Error activation (Reward win – Bivalent win) in the whole sample, thresholded at p<0.05 cluster corrected (initial cluster threshold z=2).

| **Supplementary Table 5: Clusters from whole brain analysis for Reward win minus Bivalent win in the all participants pooled, thresholded at p<0.05 cluster corrected (initial cluster z=2)** | | | | | | |
| --- | --- | --- | --- | --- | --- | --- |
| Anatomical structure | Hemisphere | Cluster size (voxels) | Peak Z-score | MNI Coordinates | | |
|  |  |  |  | x | y | z |
| Cerebellum | L/R | 3696 | 5.55 | -12 | -76 | -28 |
| DLPFC | R | 1674 | 4.63 | 46 | 32 | 30 |
|  | L | 772 | 4.03 | -50 | 22 | 34 |
| Inferior frontal gyrus | L | 1693 | 4.49 | 50 | 18 | -6 |
| Putamen | L | 502 | 3.92 | -26 | 2 | -2 |
| ACC | L/R | 835 | 4.94 | 4 | 26 | 44 |
|  |  |  |  |  |  |  |

**References**

Bernacer J, Corlett PR, Ramachandra P, McFarlane B, Turner DC, Clark L, *et al* (2013). Methamphetamine-induced disruption of Frontostriatal reward learning signals: Relation to psychotic symptoms. *Am J Psychiatry* **170**: 1326–1334.

Corlett PR, Murray GK, Honey GD, Aitken MRF, Shanks DR, Robbins TW, *et al* (2007). Disrupted prediction-error signal in psychosis: Evidence for an associative account of delusions. *Brain* **130**: 2387–2400.

Erdeniz B, Rohe T, Done J, Seidler R (2013). A simple solution for model comparison in bold imaging: the special case of reward prediction error and reward outcomes. *Front Neurosci* **7**: 116.

Jenkinson M, Beckmann CF, Behrens TEJ, Woolrich MW, Smith SM (2012). FSL. *Neuroimage* **62**: 782–790.

Martinez D, Slifstein M, Broft A, Mawlawi O, Hwang D-R, Huang Y, *et al* (2003). Imaging human mesolimbic dopamine transmission with positron emission tomography. Part II: amphetamine-induced dopamine release in the functional subdivisions of the striatum. *J Cereb Blood Flow Metab* **23**: 285–300.

Mawlawi O, Martinez D, Slifstein M, Broft A, Chatterjee R, Hwang DR, *et al* (2001). Imaging human mesolimbic dopamine transmission with positron emission tomography: I. Accuracy and precision of D(2) receptor parameter measurements in ventral striatum. *J Cereb Blood Flow Metab* **21**: 1034–57.

Murray GK, Corlett PR, Clark L, Pessiglione M, Blackwell a D, Honey G, *et al* (2008). Substantia nigra / ventral tegmental reward prediction error disruption in psychosis. *Mol Psychiatry* **13**: 1–18.

Murty VP, Shermohammed M, Smith D V, Carter RM, Huettel SA, Adcock RA (2014). Resting state networks distinguish human ventral tegmental area from substantia nigra. *Neuroimage* **100**: 580–589.

Nichols TE, Holmes AP (2002). Nonparametric permutation tests for functional neuroimaging: A primer with examples. *Hum Brain Mapp* **15**: 1–25.

Pessiglione M, Seymour B, Flandin G, Dolan RJ, Frith CD (2006). Dopamine-dependent prediction errors underpin reward-seeking behaviour in humans. *Nature* **442**: 1042–1045.

Seymour B, Daw N, Dayan P, Singer T, Dolan R (2007). Differential encoding of losses and gains in the human striatum. *J Neurosci* **27**: 4826–31.

Winkler AM, Ridgway GR, Webster MA, Smith SM, Nichols TE (2014). Permutation inference for the general linear model. *Neuroimage* **92**: 381–397.
